# Expression Pattern and Localization Dynamics of Guanine Nucleotide Exchange Factor RIC8 during Mouse Oogenesis

**DOI:** 10.1371/journal.pone.0129131

**Published:** 2015-06-10

**Authors:** Merly Saare, Sirje Lulla, Tambet Tõnissoo, Riho Meier, Keiu Kask, Katrin Ruisu, Alar Karis, Andres Salumets, Margus Pooga

**Affiliations:** 1 Institute of Molecular and Cell Biology, University of Tartu, Riia 23, 51010, Tartu, Estonia; 2 Competence Centre on Health Technologies, Tiigi 61b, 50410, Tartu, Estonia; China Agricultural University, CHINA

## Abstract

Targeting of G proteins to the cell cortex and their activation is one of the triggers of both asymmetric and symmetric cell division. Resistance to inhibitors of cholinesterase 8 (RIC8), a guanine nucleotide exchange factor, activates a certain subgroup of G protein α-subunits in a receptor independent manner. RIC8 controls the asymmetric cell division in *Caenorhabditis elegans* and *Drosophila melanogaster*, and symmetric cell division in cultured mammalian cells, where it regulates the mitotic spindle orientation. Although intensely studied in mitosis, the function of RIC8 in mammalian meiosis has remained unknown. Here we demonstrate that the expression and subcellular localization of RIC8 changes profoundly during mouse oogenesis. Immunofluorescence studies revealed that RIC8 expression is dependent on oocyte growth and cell cycle phase. During oocyte growth, RIC8 is abundantly present in cytoplasm of oocytes at primordial, primary and secondary preantral follicle stages. Later, upon oocyte maturation RIC8 also populates the germinal vesicle, its localization becomes cell cycle dependent, and it associates with chromatin and the meiotic spindle. After fertilization, RIC8 protein converges to the pronuclei and is also detectable at high levels in the nucleolus precursor bodies of both maternal and paternal pronucleus. During first cleavage of zygote RIC8 localizes in the mitotic spindle and cell cortex of forming blastomeres. In addition, we demonstrate that RIC8 co-localizes with its interaction partners Gα_i1/2_:GDP and LGN in meiotic/mitotic spindle, cell cortex and polar bodies of maturing oocytes and zygotes. Downregulation of *Ric8* by siRNA leads to interferred translocation of Gα_i1/2_ to cortical region of maturing oocytes and reduction of its levels. RIC8 is also expressed at high level in female reproductive organs e.g. oviduct. Therefore we suggest a regulatory function for RIC8 in mammalian gametogenesis and fertility.

## Introduction

Resistance to inhibitors of cholinesterase 8 (RIC8) is a guanine nucleotide exchange factor (GEF) for the α subunits of heterotrimeric G proteins [1] which was discovered during a genetic screen of *C*. *elegans* mutants that were defective in synaptic transmission [2, 3]. The RIC8 protein contains armadillo folding motifs, which are organized in a right-twisted α-super helix [4]. The functional studies have revealed that RIC8 acts as a GEF for Gα_q_, Gα_i_, Gα_o_, Gα_12_, Gα_13_ but not Gα_s_, which are activated by a paralogue of RIC8 (named RIC8B) [1, 5]. In contrast to G-protein coupled receptors (GPCRs), RIC8 interacts only with monomeric Gα subunit, participating in a non-canonic G-protein signaling pathway [1]. RIC8 associates with Gα subunits in GDP form, triggering the release of GDP and enabling binding of GTP to Gα, which disrupts the complex, resulting in free RIC8 and activated Gα-GTP [1]. However, recent findings suggest that RIC8 also has other functions, like of a molecular chaperone required for the initial targeting of nascent Gα subunits to the plasma membrane [6–8].

Two most well-known physiological functions of RIC8 were combined in its alternate name Synembryn. First its expression was shown to be restricted to different neurons of *C*. *elegans* where RIC8 plays a crucial role in regulation of synaptic signaling through G-proteins [3, 9, 10]. In mammalian cells RIC8 positively regulates Gα_q_-coupled receptor-mediated signaling and functions as a signal amplifier [1, 11]. In addition to the modulation of G-protein mediated signaling, RIC8 has been demonstrated to regulate the asymmetric cell division in different organisms. For example, it is required for the Gα_i_-mediated spindle orientation and for the acquisition of cell polarity during asymmetric division of neuroblasts and sensory precursor cells in *Drosophila* [12–14]. In early embryogenesis of *C*. *elegans* RIC8 is required for generation of proper pulling force in spindle, spindle positioning, nuclear migration and other centrosome dependent processes [15–18]. RIC8 also regulates mammalian cell division by adjusting mitotic spindle movements and positioning. More detailed studies established that RIC8 interacts with complex that contains Gα_i_-GDP, LGN and NuMA. It catalyzes dissociation of the complex to release the activated Gα_i_-GTP, NuMA and LGN, thereby regulating spindle positioning [19]. Reduction of RIC8 expression or function interferes with the localization of Gα_i_, LGN or NuMA and dynein to the cell cortex, and disrupts the correct mitotic spindle alignment in mammalian cells [20]. Recently, RIC8 was also shown to participate in growth factor induced and Gα_13_ mediated actin cytoskeleton reorganization and cell migration [21]. In mice *Ric8* homozygous mutation results in various gastrulation defects, which lead to embryonic lethality at E6.5-E8.5 [6, 22, 23].

Based on the RIC8 function to regulate the asymmetric cell division, we proposed that RIC8 might also be involved in the mammalian gametogenesis. It is well known that oocyte undergoes highly asymmetric cell divisions resulting in formation of small polar bodies and one large oocyte that contains maternal stores accumulated during oogenesis. The size difference between the daughter cells is achieved by the asymmetric spindle positioning before the cytokinesis. The female germ cells, oocytes, arise from the primordial germ cells during fetal development, as they stop dividing mitotically and enter meiosis around E13.5 [24]. Gene expression microarray analyzes in E13.5 mouse ovaries indicated that *Ric8* was upregulated at the beginning of meiosis [25]. After meiosis is initiated, primary oocytes become arrested at the diplotene stage of first prophase around the time of birth. During folliculogenesis, the oocyte grows and undergoes remodeling both on the cellular and molecular level to become fertilization-competent, and to fulfill the cellular and molecular requirements for the subsequent development. Resumption of meiosis only occurs in fully grown oocytes after the luteinizing hormone surge when oocytes undergo germinal vesicle breakdown, then they complete meiosis I and mature to metaphase II. Completion of meiosis is induced by fertilization as it triggers the progression of anaphase II, followed by the formation of 1-cell embryo that contains haploid paternal and maternal pronucleus. Recent findings have demonstrated that xRic8 (Ric8 of *Xenopus laevis*) is maternally expressed in amphibian’s oocytes where it participates in the maintenance of meiotic arrest [26, 27]. However, the role of mammalian RIC8 in these complicated processes is unknown so far.

The present study addressed the potential function of RIC8 in mammalian oogenesis by characterizing its expression and localization pattern during the oocyte growth and meiotic maturation, as well as fertilization and first zygotic cleavage process. We demonstrate that the localization of maternally expressed RIC8 protein is highly dynamic and is dependent on the stage of folliculogenesis, oogenesis and cleavage. In addition, downregulation of *Ric8* expression by siRNA in maturing oocytes leads to reduced translocation of Gα_i_ to cortical region of cells. Our findings imply that RIC8 may have a regulatory function in mammalian gametogenesis.

## Materials and Methods

### Animals

Throughout the present study wild-type C57Bl/6J mice were used. Animals were maintained under a 12 h light/12 h dark cycle and at temperature of 21°C with food and water available ad libitum. The permission for the present study was given by the Estonian National Board of Animal Experiments (Protocol Number 09 03956 from No. 9, 16 January 2009) in accordance with Directive of the Council of the European Communities of 24 November 1986 (86/609/EEC).

### Immunohistochemistry

Dissected ovaries and reproductive tracts of sacrificed adult female mice were fixed in 4% paraformaldehyde in PBS for 20 min and cryoprotected in 20% sucrose in PBS. Tissue sections with a thickness of 10 μm were cut from frozen specimens embedded in tissue freezing medium Jung (Leica Microsystems). Cryosections were mounted on Superfrost plus slides (Thermo Scientific), dried overnight, permeabilized with 0.1% Triton X-100 in PBS for 20 min and blocked for 60 min at room temperature with 1% BSA and 5% goat serum in PBS. Proteins were revealed by incubation with rabbit polyclonal anti-RIC8 (1:70, Proteintech Group, Inc) and anti-beta tubulin antibody (1:100, E7, Developmental Studies Hybridoma Bank) overnight at 4°C followed by Alexa Fluor 594 goat anti-rabbit (1:600, Invitrogen) or Alexa Fluor 633 goat anti-mouse (1:600, Invitrogen) secondary antibody for 60 min at room temperature. Cell nuclei were counterstained with DAPI and specimens mounted in Fluoromount G (Electron Microscopy Science). For negative controls, primary antibodies were omitted and no staining was observed.

### Harvesting of Oocytes and One-cell Embryos

To analyze progression of meiosis I, 4 week-old female mice were superovulated by injecting 5 IU of equine chorionic gonadotropin (eCG) and 5 IU of human chorionic gonadotropin (hCG) at an interval of 48 hours. The females were sacrificed and the ovaries were dissected in 4 to 10 hours after hCG injection (taken at hourly intervals). Oocytes were harvested by puncturing ovarian follicles with sterile needle. Surrounding cumulus cells were removed with hyaluronidase (0.3 mg/ml, Sigma Aldrich) solution by gentle pipetting.

To study meiosis II and first zygotic division, female mice at age of 10–11 weeks were mated. Fertilized oocytes were harvested from the oviducts at approximately 40 min to 20 h after the detection of the vaginal plug and were treated with hyaluronidase.

### Harvesting of Oocytes and Microinjection

4 week-old female mice were superovulated by injecting 5 IU of eCG. The females were sacrificed and ovaries were dissected in 48 hours after eCG injection. Ovaries were placed in M2 medium (Sigma) and punctured several times with sewing needles that are fastened together. Oocytes were collected and cumulus cells were removed by pipetting several times. Cumulus free oocytes were transferred to KSOM medium (Millipore) that contained 0,2 mM 3-isobutyl-1-methylxanthine (IBMX) (Sigma), an inhibitor of cyclic nucleotide phosphodiesterase, which prevents the resumption of meiosis. After 2 h recovery period, the oocytes were microinjected with 2–10 pl of mouse *Ric8* siRNA (20 μM) (ON-TAGRGETplus SMART pool, Dharmacon) or non-targeting pool (20 μM) (ON-TAGRGETplus Control pool, Dharmacon). Next, the oocytes were incubated for 22 h in KSOM+IBMX medium, washed 3 times with M2 medium and matured *in vitro* in KSOM medium for 24 h at 37°C in CO_2_ incubator. Oocytes were washed with PBS and in each group 10 oocytes were taken for cDNA synthesis that was performed according to manufacturers protocol (SuperScript III CellsDirect cDNA synthesis System, Invitrogen). Remaining oocytes were fixed in 4% paraformaldehyde in PBS and used for immunocytochemistry.

### Immunocytochemistry

Oocytes and one-cell embryos were fixed in 4% paraformaldehyde in PBS for 15 min at room temperature, permeabilized with 0.2% Triton X-100 for 20 min and blocked with 1% BSA in PBS for 1 h. Oocytes were stained as described above incubating with rabbit polyclonal anti-RIC8 antibody for 4 h at room temperature and using secondary antibody at dilution 1:800. Microfilaments were revealed with Alexa Fluor 488 phalloidin (1:125, Invitrogen), applied along with secondary antibodies.

For co-localization experiments, after staining with goat polyclonal RIC8 antibody (1:30; secondary antibody Alexa Flour 488 donkey anti-goat 1:800) and washes in PBS, oocytes were again blocked in 1% BSA-supplemented PBS for 1 h at room temperature, stained with rabbit polyclonal anti-NuMA (1:100, Abcam), rabbit polyclonal anti-LGN (1:100, Abcam) or mouse monoclonal anti-β-tubulin antibody for 4 h at room temperature. After washes in PBS, secondary antibody Alexa Fluor 555 goat anti-rabbit (1:800, Invitrogen) or Alexa Fluor 633 goat anti-mouse (1:800, Invitrogen) was added. After washes in PBS, oocytes were stained with DAPI (Sigma Aldrich) and mounted with Floromount (EMS). For negative controls primary antibodies were omitted and no staining was observed.

### Quantitative RT-PCR

Quantitative RT-PCR (using Life Technologies Applied Biosystems StepOnePlus Real-Time PCR instrument) was performed using cDNA from oocytes. The level of *Ric8* downregulation was estimated from 3 separate siRNA injection experiments using 10 oocytes per experiment. The reaction was carried out for 40 cycles of 15 seconds at 95°C and 1 minute at 60°C in an optimized ready-to-use solution (HOT FIREPol EvaGreen qPCR Mix Plus, Solis BioDyne, Estonia). Constitutively expressed housekeeping gene *Gapdh* (*Glyceraldehyde 3-phosphate dehydrogenase*) was chosen as reference using 5′-CACAGGACTAGAACACCTGC-3′ and 5′-GCTGGTGAAAAGGACCTCT-3′ primers. For relative *Ric8* mRNA expression was analysed by using primers 5′-GAGGAGTTCCACGGCCACA-3′ and 5′-CTTCAGCCTGTGGGTCTGGTG-3′. The relative *Ric8* expression was calculated using ΔΔCt method.

### Confocal Microscopy

Images were captured by Olympus IX81 inverted microscope equipped with the FluoView FV1000 confocal system, using excitation at 405 nm (DAPI), 488 nm (Alexa Flour 488), 559 nm (Alexa Flour 594 or 555) and 635 nm (Alexa Flour 633) and analyzed by Olympus FV1000 software. For Alexa Flour 594 or 555 and Alexa Flour 633 lasers were run in sequential mode to avoid the spectral overlap. Images were processed with Adobe Photoshop CS4.

### Quantification of RIC8 and Gα_i1/2_ protein in maturing oocyte

Fluorescence intensity estimation was used for quantification of RIC8 and Gα_i1/2_ expression in oocyte cortex and cytoplasm by using AutoQuant X3 (MediaCybrnetics, INC). Average intensity of RIC8 (in control n = 11 and in *Ric8* siRNA n = 21) and Gα_i1/2_ (in control n = 7 and in *Ric8* siRNA n = 8) proteins signal was measured in the cell cortex and cytoplasm of three equatorial confocal sections of each cell. To determine the relative expression level of RIC8 and Gα_i1/2_ protein, the average fluorescence intensity was normalized to the selected surface area. The presented results show the relative level of protein per unit area.

### Statistical analysis

Student *t* test was used to estimate the differences between groups (parametric analysis). The differences of *P*˂0.05 were considered significant. Results were expressed as mean ±SEM.

## Results

### Subcellular Localization of RIC8 During Folliculogenesis and in the Reproductive Tract

**Fig 1:**
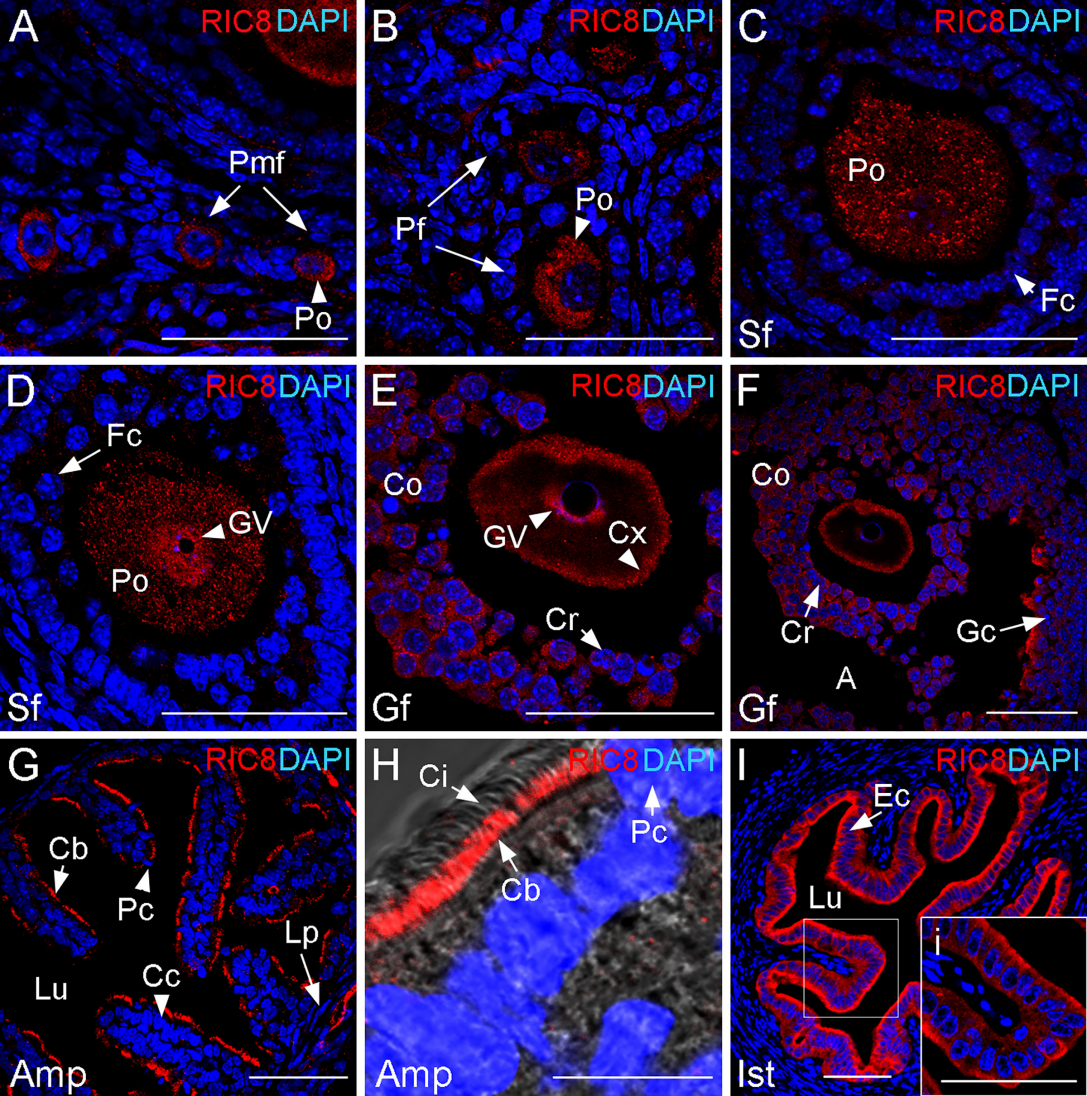
RIC8 in folliculogenesis and in the reproductive tract of adult mouse. RIC8 was visualized with RIC8 antibody (red), and cell nuclei were visualized with DAPI (blue). (**A-F**) Transversal cryosections of ovary with oocytes in different follicular stages starting from primordial follicle to Graafian follicle and (**G-I**) different regions of oviduct are shown. (**H**) Higher magnification of the region of ampulla and (**i**, indicated by white box) isthmus. Abbreviations: A, antrum; Amp, ampulla region of oviduct; Cb, basal layer of cilia; Cc, ciliated cell; Ci, cilia; Co, cumulus oophorus; Cr, corona radiata; Cx, cell cortex, Ec, epithelial cells; Fc, follicular cell; Gc, granulosa cells; Gf, Graafian follicle; GV, germinal vesicle; Ist, isthmus region of oviduct; Lp, lamina propria; Lu, lumen; Pc, peg cell; Pf, primary follicle; Pmf, primordial follicle; Po, primary oocyte; Sf, secondary follicle. Scale bars: 50 μm.

The dynamics of RIC8 localization during mitosis in the cultured mammalian cells was characterized recently [20], but its expression pattern in the mammalian meiosis has not been assessed so far. Therefore, we analyzed RIC8 localization in the course of oogenesis in adult mouse until fertilization by using immunohistochemistry. Mammalian oocytes are arrested at the diplotene stage of the first meiotic prophase. During the process of folliculogenesis, oocytes enter the growth phase and finally resume meiosis. At the primordial (Fig 1A) and primary follicle stage, when the oocyte enters the growth phase (Fig 1B), RIC8 is localized to the cytoplasm of the primary oocyte. At the later preantral and antral secondary follicle stages, RIC8 retains its cytoplasmic localization (Fig 1C and 1D). However, at the antral secondary follicle stage, when the chromatin starts to fold into surrounded nucleolus (SN) configuration [28], RIC8 is also targeted to germinal vesicle (Fig 1D). At the preovulatory stage, when the chromatin is fully condensed around the nucleolus, in the SN configuration, RIC8 localizes in the germinal vesicle. In parallel RIC8 accumulates from cytoplasm to the cell cortex (Fig 1E and 1F). RIC8 is also present at detectable level in the cytoplasm of the surrounding cumulus cells and mural granulosa cells (Fig 1E and 1F).

To assess the expression and localization of RIC8 protein in the female reproductive tract we analyzed the cross-sections from different areas of the Fallopian tube and uterus by immunohistochemistry. The oviduct epithelium of adult female mouse consists of two types of epithelial cells, which are unevenly distributed. Ciliated cells are predominant in the region of infundibulum and ampulla, and secretory cells in the isthmus. RIC8 was present in all regions of the Fallopian tube. More precisely, in the area of ampulla, it was clearly localized in the basal layer of the cilia of the ciliated cells (Fig 1G and 1H). RIC8 was also detectable at high level in the epithelium of the isthmus. Interestingly, the localization of RIC8 seemed to be polarized as it accumulated more in the apical cell cortex than in basal (Fig 1I and 1i). RIC8 expression was also detectable in the endometrium of uterus but at the markedly lower level (data not shown).

### During Oocyte Maturation RIC8 Accumulates on Chromatin and Meiotic Spindle

The final phase of folliculogenesis comprises the resumption of meiosis and germinal vesicle breakdown, which are triggered by a surge of luteinizing hormone. In order to map the RIC8 protein localization after meiotic resumption, we characterized the oocytes at different stages of meiosis I, and in *in vivo* fertilized oocytes during meiosis II by using immunocytochemical analysis with RIC8 antibody.

**Fig 2:**
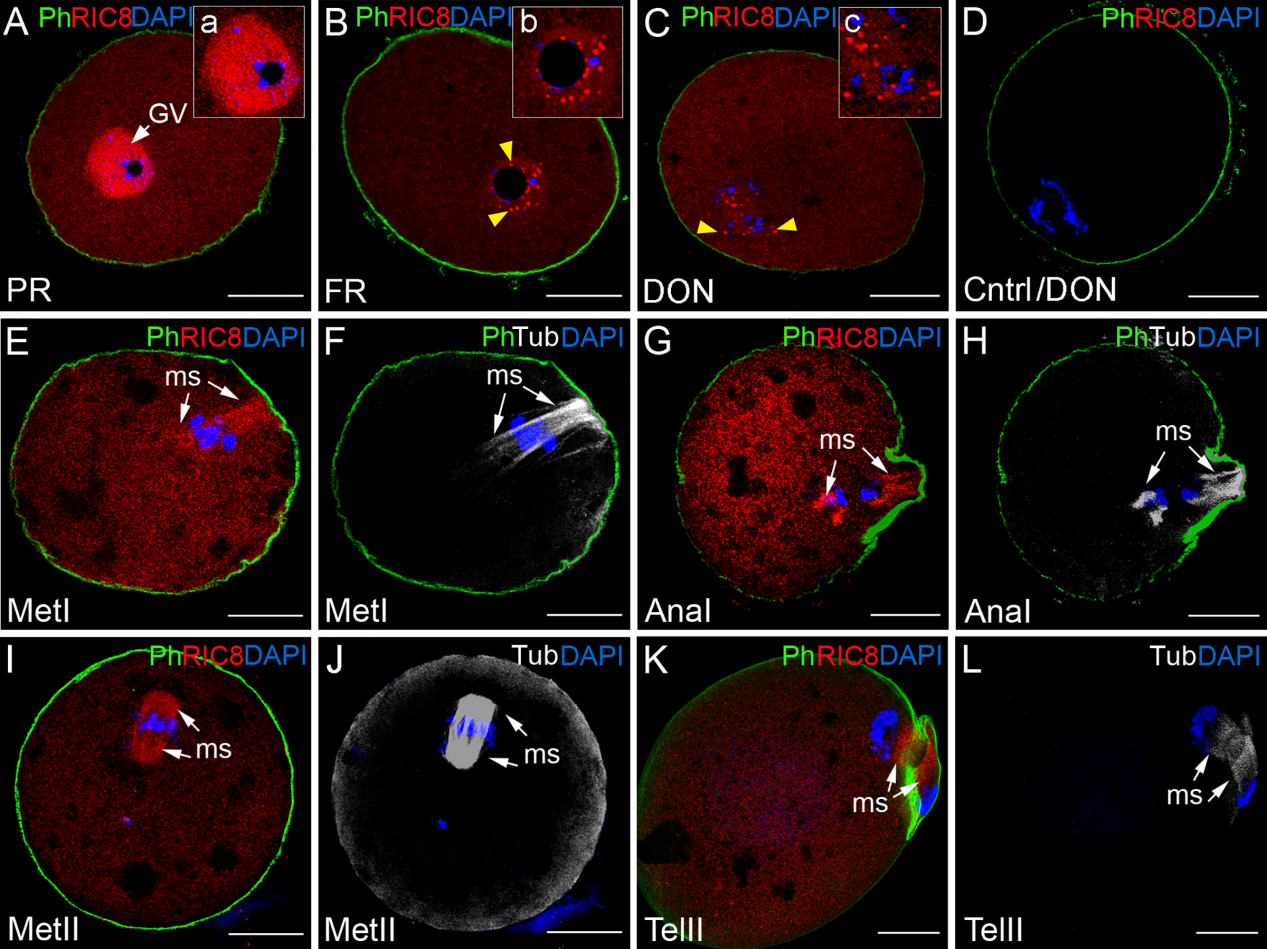
Dynamic changes in localization of RIC8 in the mouse oocyte during meiosis. RIC8 was visualized with RIC8 antibody (red), microtubules with antibody to β–tubulin (Tub, white), chromatin with DAPI (blue) and F-actin with phalloidin (Ph, green). (**A, B**) GV- stage oocyte; (**C, D**) GVBD (germinal vesicle breakdown) stage oocytes; (**E-H**) oocytes undergoing meiosis I or (**I-L**) meiosis II. (**a`**, **b`**, **c`**) Higher magnification of the region of chromatin. Yellow arrowheads indicate the RIC8 foci at nucleolus and on chromatin. White arrows point to the meiotic spindle. (**D**) Negative control. Abbreviations: AnaI, anaphase of meiosis I; DON, disappearance of the nucleolus; GV, germinal vesicle; FR, full rim stage; MetI/MetII, metaphase of meiosis I or II; ms, meiotic spindle; PR, partial rim stage; TelII, telophase of meiosis II. Scale bar: 20 μm.

**Fig 3:**
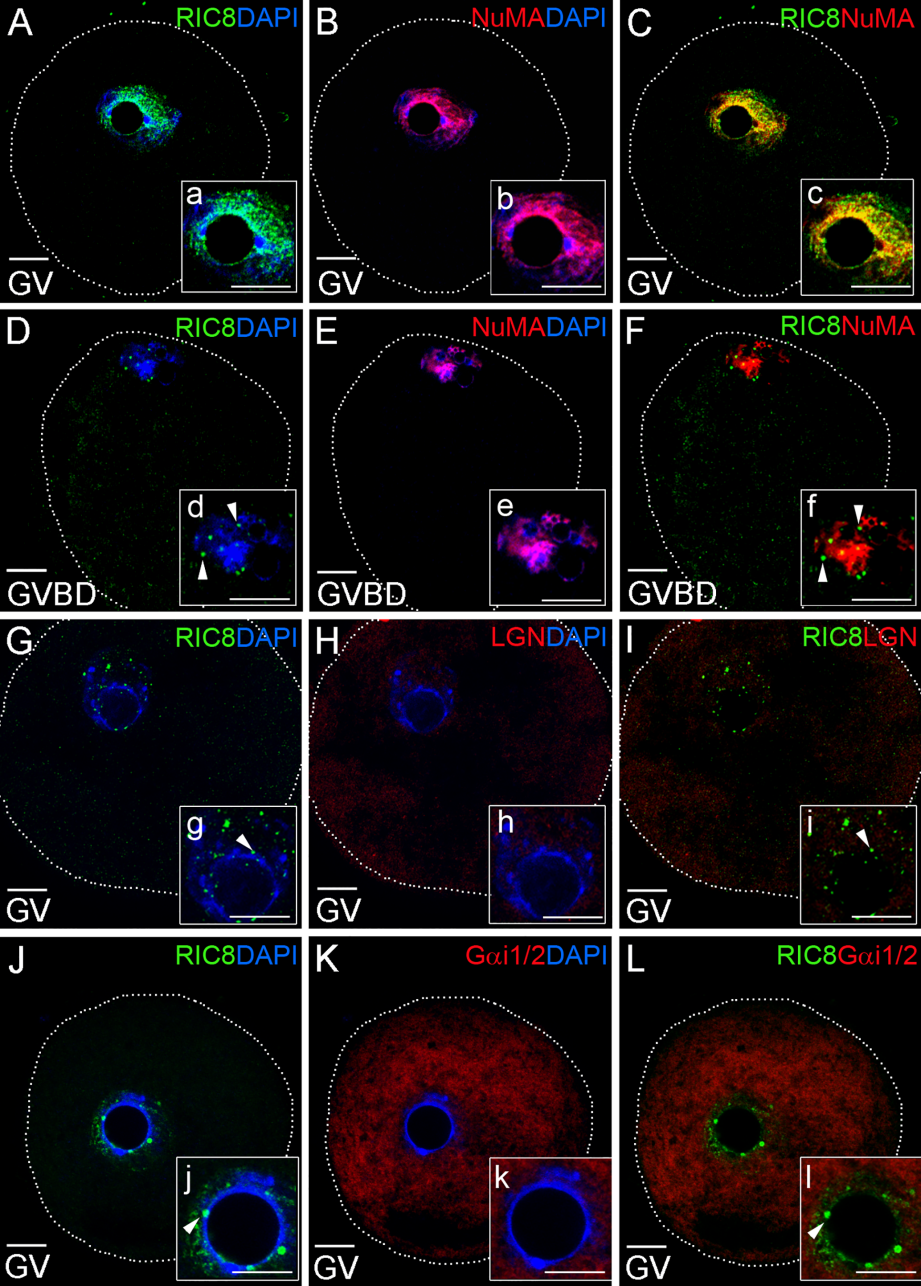
Immunological detection of co-localization of RIC8 with Gα_i1/2_, LGN and NuMA proteins during oocyte development. (**A-C**; **G-L**) Mouse oocytes at GV or (**D-F**) GVBD stages were double-labeled with RIC8 antibody (green) and NuMA, LGN or Gα_i1/2_ antibodies respectively (red). DNA was stained with DAPI (blue). (**a-l**) Higher magnification of the region of chromatin. Dotted white line indicates the borders of oocyte. White arrowheads point RIC8 foci at nucleolus and on chromatin. Abbreviations: GV, germinal vesicle; GVBD, germinal vesicle breakdown. Scale bar: 10 μm.

**Fig 4:**
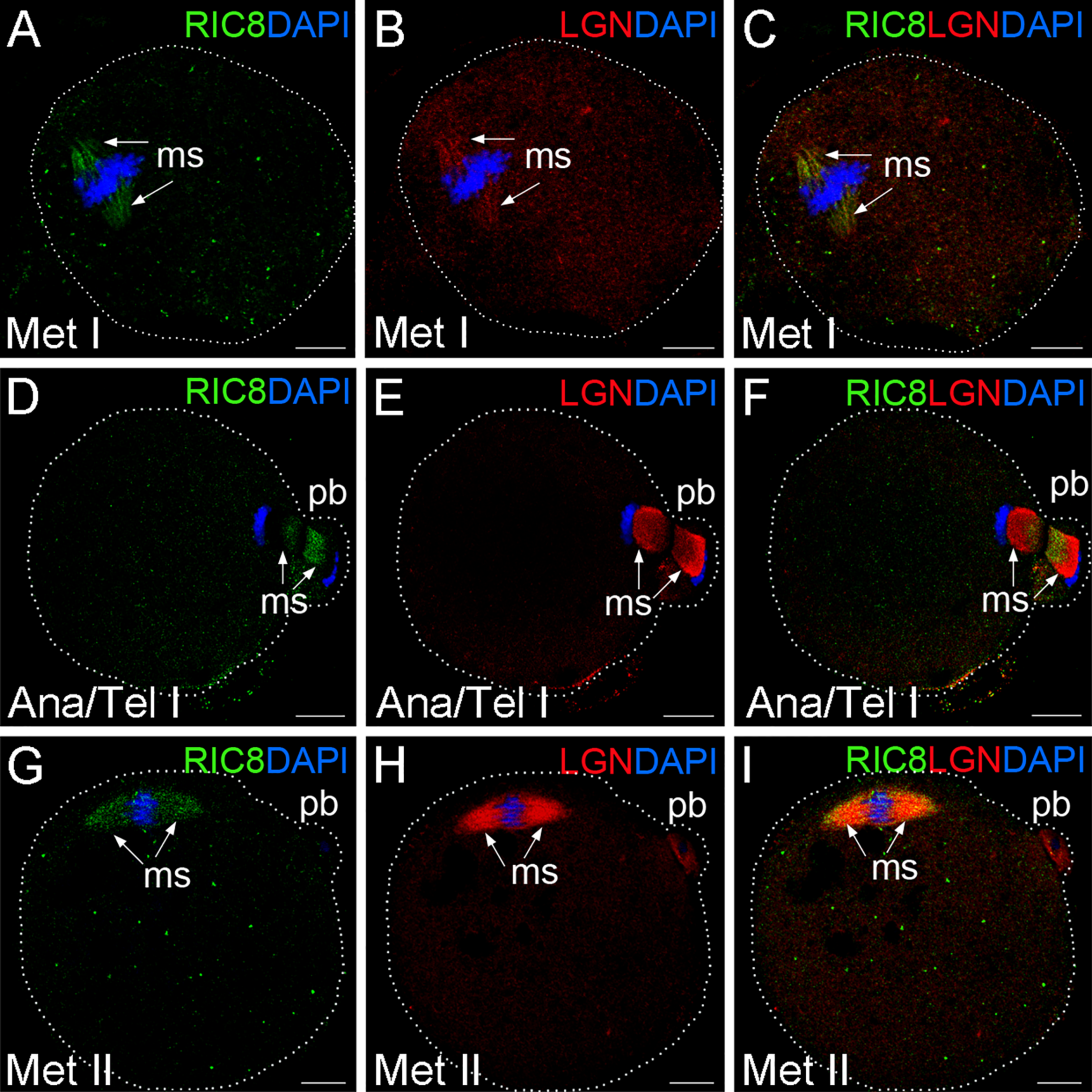
GEF RIC8 co-localization with LGN during mouse oocyte maturation. (**A-F**) Mouse oocytes at meiosis I and (**G-I**) at metaphase block of meiosis II were double-labeled with RIC8 antibody (green) and LGN (red). DNA was stained with DAPI (blue). Localization of meiotic spindle is denoted with white arrows and yellow to orange colour in this area indicates the overlapping regions of RIC8 and LGN. Dotted white line indicates the borders of oocyte. Abbreviations: Ana/Tel I, anaphase/telophase of meiosis I; Met I or Met II, metaphase of meiosis I or meiosis II respectively; ms, meiotic spindle; pb, polar body. Scale bar: 10 μm.

**Fig 5:**
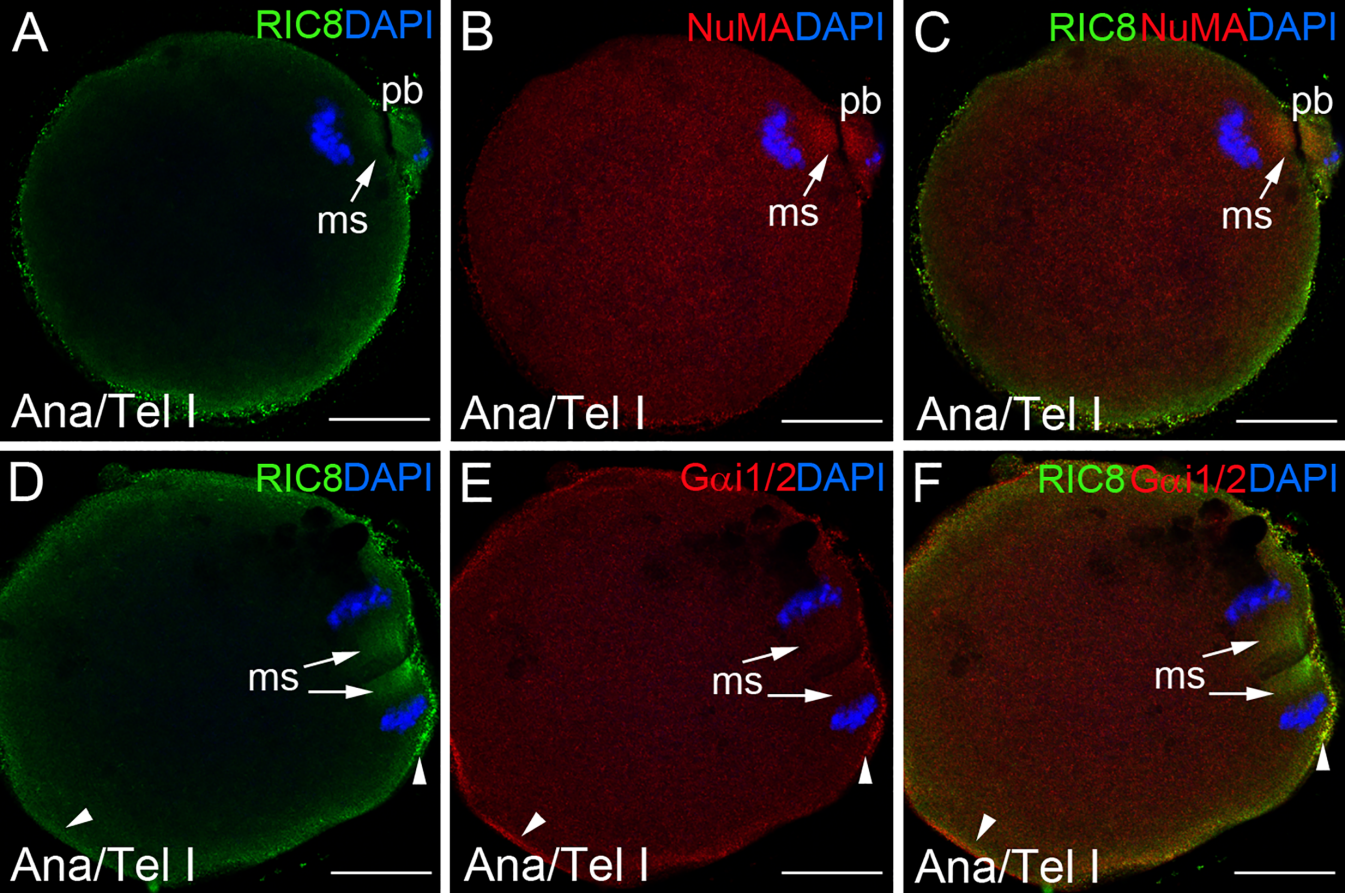
RIC8 protein expression pattern compared to NuMa or Gα_i1/2_ proteins during mouse oocyte maturation. (**A-F**) Mouse oocytes at meiosis I were double-labeled with RIC8 antibody (green) and NuMA or Gα_i1/2_ antibodies (red). DNA was stained with DAPI (blue). The overlapping regions of RIC8 and NuMA or Gα_i1/2_ (yellow to orange) at meiotic spindle indicated with white arrows. White arrowheads indicated cell cortex regions, where RIC8 and Gα_i1/2_ co-localize. Abbreviations: Ana/Tel I; anaphase/telophase of meiosis I; ms, meiotic spindle; pb, polar body. Scale bar: 10 μm.

Before the germinal vesicle breakdown, dynamic changes take place in the chromatin configuration of germinal vesicle, which are associated with transcriptional activity. The localization of RIC8 was assessed at different maturation stages of nucleus, which were assigned based on the chromatin configuration. First, we discovered that when the chromatin started to condense and it was identified as a partial rim (PR) around the nucleolus, RIC8 remained diffusely localized across the germinal vesicle (Figs 2A, 2a and 3A), which was also typical for the secondary follicle stage in the ovary (Fig 1D). Later, at the full rim [28] stage, when chromatin had condensed densely around nucleolus, RIC8 accumulated in spots distributed around condensed chromatin in the germinal vesicle, with some foci attached to the nucleolus (Figs 2B, 2b, 3G and 3J). Further, when the nuclear lamina as well as the nucleolus started to disappear, the spots of RIC8 remained in the close vicinity of chromosomes (Figs 2C, 2c, 3D and 3d). With the organization of chromosomes to the metaphase plate, RIC8 distributed along the metaphase spindle (Figs 2E and 4A) and co-localized with tubulin (Fig 2E and 2F). In anaphase I, during the chromosome separation, RIC8 remained in the vicinity of chromosomes and co-localized with the meiotic spindle (Figs 2G, 2H and 4D). After completion of meiosis I, the secondary oocytes enter directly meiosis II, at which point they are arrested for the second time, as the chromosomes move to the metaphase II plate. During the second arrest, RIC8 was distributed to the metaphase spindle and diffusely in the cytoplasm (Fig 4G). After the ovulation, fertilization triggers the resumption and completion of meiosis II and metaphase is rapidly finished. During the final steps of meiosis II RIC8 was located in the spindle (Fig 2I–2L). In addition to the above-mentioned, a part of RIC8 protein was also detectable uniformly in the cytoplasm of oocyte at every examined stage, and also in some extent at the cell cortex (Fig 5A and 5D).

### RIC8 co-localizes with NuMA in the germinal vesicle of oocyte

Earlier biochemical studies have indicated that RIC8 acts on a ternary complex of Gα_i_-GDP, LGN (mammalian Pins homolog) and NuMa (nuclear mitotic apparatus protein) inducing the release of Gα_i_-GTP and dissociation of NuMa from LGN, which may promote the pulling of aster microtubules during cell division [19]. Furthermore, RIC8 is necessary for the assembly of a cortical signaling complex that orients the mitotic spindle during mammalian cell division [20]. In order to characterize RIC8, Gα_i1/2_, LGN and NuMA, during mammalian oogenesis, meiosis and fertilization, we examined whether these proteins were co-localized at afore-mentioned stages. By using immunocytochemical studies, we found that at germinal vesicle (GV) stage of oocyte maturation RIC8 (Fig 3A and 3a) and NuMA ([29, 30]; Fig 3B and 3b) were both broadly expressed in GV and their localization partially overlapped (Fig 3C and 3c). After germinal vesicle breakdown (GVBD) NuMA and RIC8 were both starting to condense around chromatin but NuMA expression was much wider (Fig 3E and 3e) compared to RIC8, which had accumulated as discrete spots near chromatin (Fig 3D and 3d). As expected, LGN was weakly detectable in cytoplasm at GV and GVBD stages but was congregated around chromatin during prometaphase ([31]; Fig 3H and 3h). However, co-localization of RIC8 and LGN in GV or GVBD stages was not detected (Fig 3G–3I). Gα_i1/2_, as one of the direct interaction partners of RIC8, was uniformly distributed in cytoplasm of oocyte at GV and GVBD stages, and did not reveal significantly overlapping localization with RIC8 (Fig 3J–3L).

### RIC8 exhibits similar localization pattern with NuMa, LGN and Gα_i1/2_ in meiotic spindle

Next, we analyzed the cellular localization of RIC8 protein in relation to its interaction partner Gα_i1/2_ as well as LGN and NuMA at different meiotic stages of oocyte development. At metaphase I RIC8 and LGN showed high extent co-localization at the meiotic spindle apparatus (Fig 4A–4C) and they both remained associated with spindle during anaphase and telophase ([31]; Fig 4D–4F). However, the degree of their co-localization in this area decreased as LGN localized more close to chromosomes (Fig 4E), whereas RIC8 condensed more at the plus-end of meiotic spindle (Fig 4D). Later, in MII metaphase arrested oocytes the extent of RIC8 and LGN co-localization was similar to MI oocytes (Fig 4G–4I).

The locaton of another regulatory protein NuMA partially overlaps with RIC8 in the spindle during meiosis I. In the anaphase and telophase, when chromosomes segregate, NuMA and RIC8 translocated to the spindle midzone (Fig 5A–5C), whereas Gα_i1/2_ remained in the cortex area during meiosis I and II (Fig 5E and data not shown for meiosis II). In parallel with accumulation in the meiotic spindle RIC8 also localized to the cell cortex in a partially overlapping manner with Gα_i1/2_ (Fig 5D–5F). Gα_i1/2_, on the other hand, was only weakly detectable in the meiotic spindle as compared to RIC8, which was rather abundant in this region (Fig 5D–5F).

### After Fertilization RIC8 Translocates to the Pronuclei

**Fig 6:**
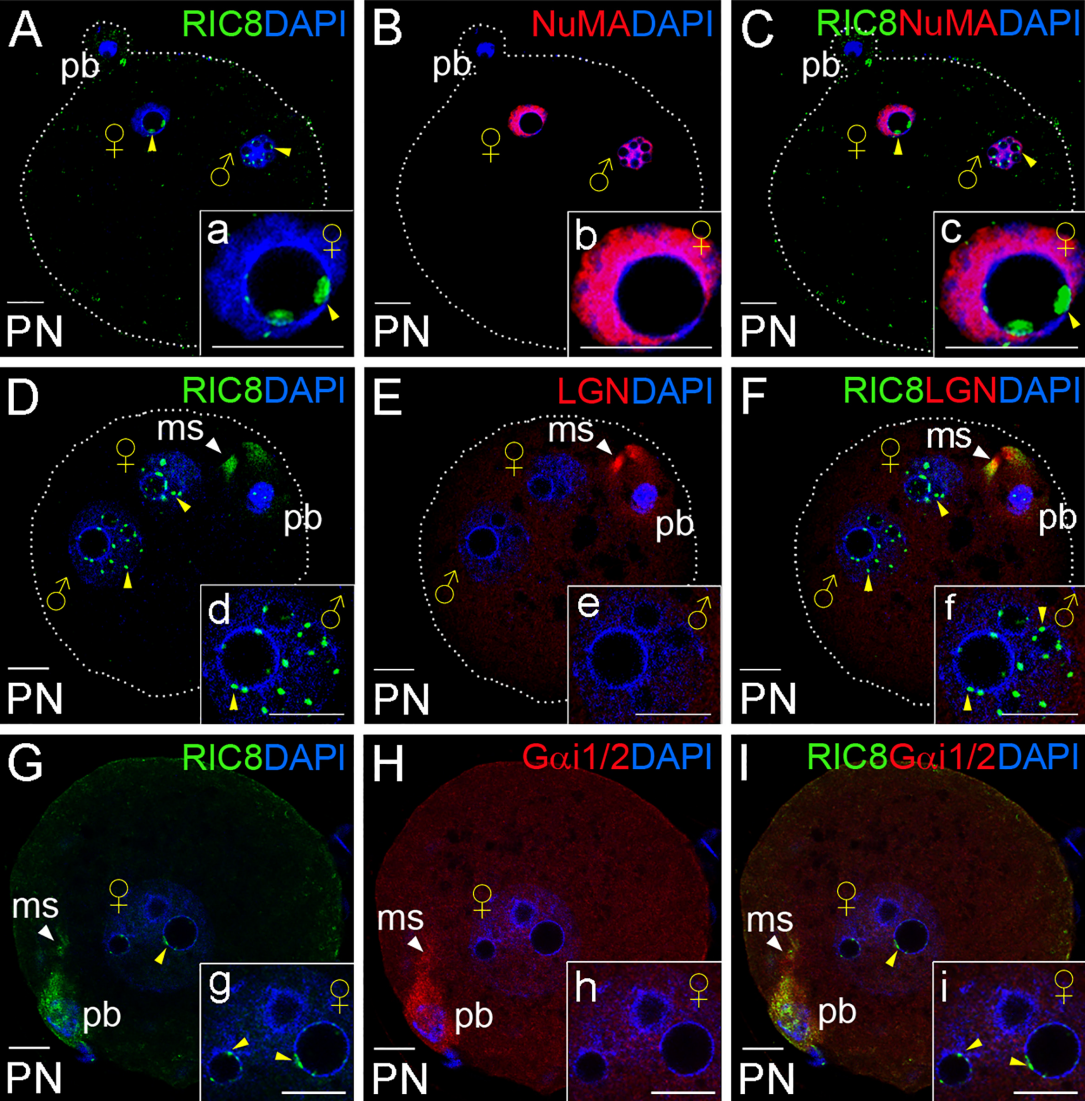
Distribution of RIC8, Gα_i1/2_, LGN and NuMA in the fertilized oocyte at pronuclear stages. (**A-I**) Mouse fertilized oocyte at pronuclear stages were double-labeled with RIC8 antibody (green) and NuMA, LGN or Gα_i1/2_ antibodies respectively (red). DNA was stained with DAPI (blue). (**a-i**) Higher magnification of female or male (indicated by sex symbols) pronucleus. Yellow arrowheads point to small RIC8 foci localized in the nucleoplasm. White arrowheads point to the meiotic spindle. Dotted white line indicates the borders of oocyte. Abbreviations: ms, meiotic spindle; pb, polar body; PN, pronuclear stage. Scale bar: 10 μm.

The oocyte liberates from the metaphase II arrest after a sperm penetration and resumes to meiosis II. The final stages of oocyte meiosis take place in parallel with the transport of sperm’s nuclear material (male pronucleus) towards the forming female pronucleus. After the sperm penetration and the completion of meiosis II, RIC8 converged into the traveling male pronucleus and the forming female pronucleus. RIC8 accumulated strongly in the nucleolus precursor bodies (NPBs), the morphological intermediates of reforming nucleolus (Fig 6A and 6a). Notably, also the nucleoli of the second polar body contained RIC8 protein at high level (Fig 6D). In addition, we examined the possible co-localization of Gα_i1/2_, NuMA and LGN proteins at pronuclear stages with RIC8, and we found that RIC8 was localized in the pronucleus as discrete spots around the nucleoli (Fig 6A and 6D). Although NuMA was broadly localized to the pronucleus (Fig 6B and 6b), it showed no overlap with RIC8 (Fig 6C and 6c). LGN, in contrary, was not detectable in pronuclei (Fig 6E and 6e) although it was discernible in the remains of spindle of the second polar body, where it co-localized with RIC8. Interestingly, LGN and RIC8 co-localized in the middle area of spindle, but did not overlap at the plus or minus ends of spindle (Fig 6F and 6f). Moreover, Gα_i1/2_ exhibited similar localization pattern in the remnants of spindle with LGN, although it was also detectable in the cell cortex and polar body in analogy with RIC8 (Fig 6H).

### Dynamics of the Subcellular Localization of RIC8 in Early Cleavage

**Fig 7:**
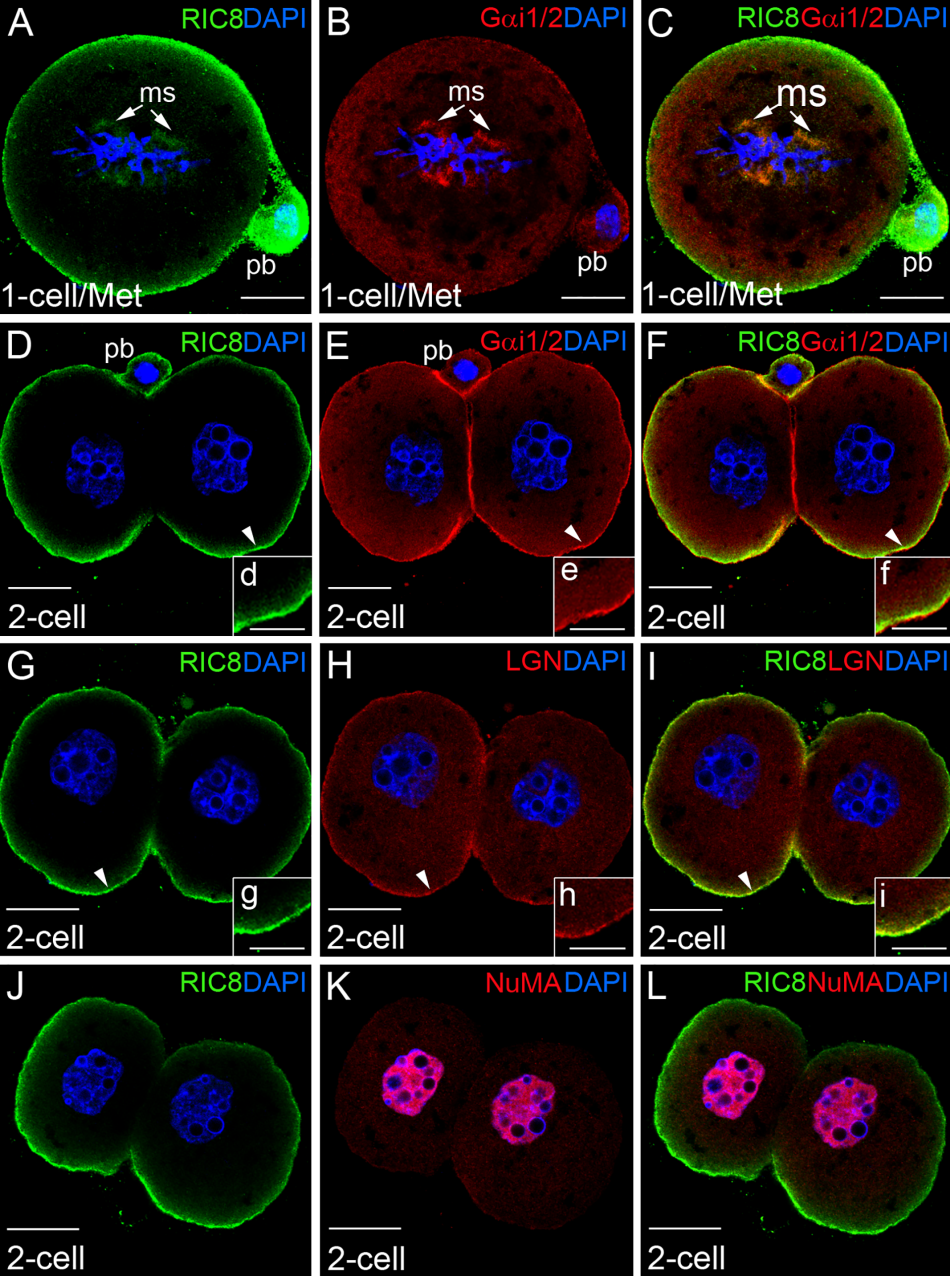
Localization pattern of RIC8, Gα_i1/2_, LGN and NuMA at early cleavage stage of mouse embryo. (**A-C**) One-cell (1-cell) embryo at metaphase (Met) of first mitosis. (**D-L**) Two-cell (2-cell) mouse embryos. Embryos were double-labeled with RIC8 antibody (green) and Gα_i1/2_, LGN or NuMA antibodies respectively (red). DNA was stained with DAPI (blue). (**d-i**) Higher magnification of overlapping regions (yellow to orange) of RIC8 and Gα_i1/2_ or LGN in cortex area of blastomere (indicated with white arrowhead). Abbreviations: ms, mitotic spindle; pb, polar body. Scale bar: (**A-L**) 20 μm, (**d-i**) 10 μm.

To elucidate the localization of RIC8 protein during early cleavage we analyzed zygote development until two-cell stage by immunocytochemistry. During the metaphase of the first mitosis, RIC8 had shifted to the metaphase plate in the close vicinity of chromosomes and microtubules, and also co-localized with Gα_i1/2_ and LGN in mitotic spindle and cell cortex (Fig 7A–7C, data not shown for LGN). After the first mitotic cell division, when the formed blastomeres still adhere to each other, RIC8 is localized in the apical region of the cell cortex with specific exclusion from the region of cell-cell contact (Fig 7D, 7G and 7J). Although, in the cell cortex RIC8 typically co-localized with Gα_i1/2_ (Fig 7B–7C and 7E–7F) and LGN (Fig 7H and 7I); surprisingly, in the region between blastomeres Gα_i1/2_ yielded a strong and LGN weak immunofluorescence signal in contrary to RIC8, which was clearly absent in this area (Fig 7E *vs* 7H). A closer inspection of the apical cortex region of blastomeres, by using higher magnification, indicated that RIC8 and LGN localization overlapped more extensively (Fig 7g–7i) than that of RIC8 and Gα_i1/2_ in the same region (Fig 7d–7f). In addition, we found no co-localization between RIC8 and NuMA in blastomeres as NuMA was present in the nucleus and RIC8 in the cortex region (Fig 7J–7L).

### Downregulation of RIC8 in maturing oocyte interferes with the Gα_i1/2_ localization in the cell cortex

**Fig 8:**
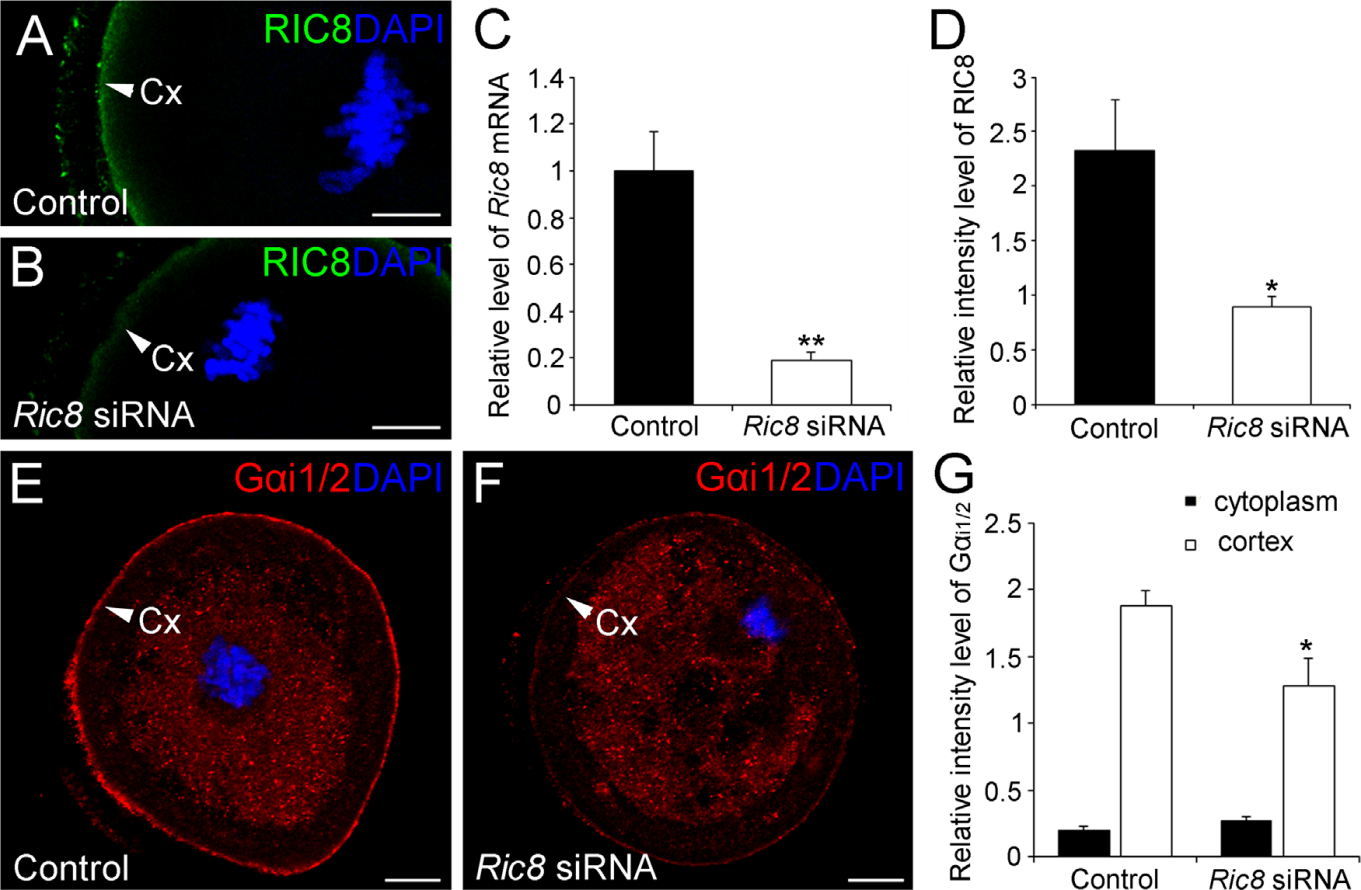
RIC8 downregulation interferes with localization of Gα_i1/2_ protein to the oocyte cortex. (**A, B**) RIC8 protein (green) expression in mouse oocytes was downregulated by microinjection of *Ric8* siRNA. DNA was stained with DAPI (blue). (**C**) Downregulation of *Ric8* mRNA expression with siRNA in microinjected oocytes quantified by qRT-PCR. (**D**) The relative intensity of RIC8 immunofluorescence signal measured by AutoQuant X3. (**E, F**) Localization of Gα_i1/2_ (red) in mouse oocytes treated with *Ric8* and control siRNA at meiosis I. DNA was stained with DAPI (blue). (**G**) The relative intensity of Gα_i1/2_ immunofluorescence signal measured by AutoQuant X3. Abbreviations: Cx, cortex. *P ˂ 0.05, **P ˂ 0.01; Students *t* test. Error Bars represent ±SEM scores. Scale bar: 10 μm.

In order to explore the function of RIC8 in the maturation process of mouse oocyte, we downregulated the endogenous expression of *Ric8* mRNA by siRNA. The quantitative real-time PCR revealed a strong reduction (80%; *P*<0.01) in the relative level of *Ric8* mRNA in siRNA treated oocytes compared to controls (non-targeting siRNA) (Fig 8C). To assess the changes in RIC8 protein expression we quantified the immunofluorescence signal intensity of RIC8 in specimens of maturing oocytes in both cells groups (oocytes treated with non-targeting siRNA or *Ric8* targeting siRNA). We found that the RIC8 signal intensity in *Ric8* siRNA treated oocytes was sigificantly lower (*P*<0.05; 61.7%) in the cortex region of oocytes as compared to controls (Fig 8A, 8B and 8D), but in the cytoplasm and the meiotic spindles the differences between groups were not discernible (*P* = 0.60) (data not shown). These results indicate that *Ric8* siRNA efficiently dowregulated *Ric8* expression and also RIC8 protein level. Moreover, we also observed that for *Ric8* siRNA oocytes maturation from GV-stage to meiosis II metaphase took more time than for the control group. In order to clarify whether downregulation of RIC8 also affects the localization of its interaction partners LGN and Gα_i1/2_ in maturing oocytes, we performed immunofluorescence analysis. The localization pattern of LGN (data not shown) and Gα_i1/2_ was not influenced by *Ric8* siRNA treatment of oocytes, but we noticed that the accumulation of Gα_i1/2_ in the cortex region was lower than in control oocytes (Fig 8E and 8F). Quantification of the Gα_i1/2_ immunofluorescence signal of did not reveal any differences in Gα_i1/2_ levels in the cytoplasm region (*P* = 0.14; Fig 8G) between the *Ric8* siRNA treated and control oocytes. However, the cortical signal of Gα_i1/2_ was significantly lower in *Ric8* siRNA treated oocytes compared to control ones (*P*<0.05; Fig 8G). Therefore, our results indicate that RIC8 is required for the maintenance of Gα_i1/2_ levels and its targeting to the cortical region of maturating oocytes.

## Discussion

Microarray analysis of mouse ovaries, at embryonic day 13.5 when oocytes stop dividing mitotically and enter meiosis [24] revealed that the expression of *Ric8* gene was upregulated with the onset of meiosis [25]. In order to assess the function of RIC8 in later steps of oogenesis, in meiosis, we mapped its localization during mouse oocyte growth and maturation, fertilization and initial cleavage steps. We demonstrate that at the earlier stages—primary oocytes at the diplotene of the first meiotic prophase and during folliculogenesis until preantral follicle stages RIC8 localizes in the cytoplasm. However, at later stages of folliculogenesis, RIC8 also accumulates to the germinal vesicle at the partial rim stage. When chromatin condenses around the nucleolus at the full rim stage, RIC8 surrounds chromatin in the germinal vesicle as characteristic spots. The localization of RIC8 in specific foci is reminiscent of centromere pattern [32], and in support with that RIC8 has been shown to co-localize with centromeres in HeLa cells [20]. Upon meiotic spindle formation RIC8 shifted to the spindle in metaphase and remained there during the anaphase and telophase of meiosis I and II. At the initial steps of oocyte first cleavage, RIC8 accumulated in the region of chromosomes and microtubules, and in the cell cortex. Interestingly, at two-cell stage where interaction with β-catenin and E-cadherin mediate adhesion of the blastomeres [33], RIC8 was specifically excluded from the cell cortex between blastomeres. In analogy, RIC8 is shown to localize to the cortex, spindle poles, centromeres, central spindle and midbody in HeLa cells depending on the phase of cell cycle [20]. Rather similar pattern of RIC8 localization and redistribution has been observed in various model organisms. For example in the early *C*. *elegans* embryo it has been mapped in the cell cortex and on the asters of the mitotic spindle, but also on the central spindle, at the nuclear envelope, around the chromatin, and at the junctions between cells [15, 17, 34]. Analogously, in *Drosophila* Ric-8 localizes in cytoplasm of NB (neuroblasts) and pI cells (sensory precursor cells) and concentrates at the mitotic spindle [13, 14].

Interestingly, immunoprecipitation of chromosomal passenger complex (CPC) revealed that RIC8 is involved in or coupled to CPC. Remarkably, in the CPC RIC8 was phosphorylated, as shown by mass spectrometry and reaction with phosphorylation specific antibody P190, implying that the cellular localization of RIC8 is controlled by phosphorylation [35]. Furthermore, it was recently shown that aPKCλ is required for the phosphorylation of RIC8 at Ser501, which could control RIC8 subcellular localization [36]. The meiotic CPC has analogous function with mitotic CPC, correcting chromosome attachment to microtubules, facilitating the spindle-assembly checkpoint function and enabling cytokinesis [37]. Interaction partners within CPC have been mapped only in the case of xRic8 (Ric8 of *Xenopus laevis*) which associates with DasRa and INCENP in complex [4]. However, the function of RIC8 in the CPC and the function or molecular mechanism of RIC8 in meiosis has not been defined yet. Still, its localization pattern during oocyte maturation resembles to the positioning of RIC8 in mitosis, strongly suggesting that the basic function of RIC8 is the same in both processes.

In dividing mammalian cells RIC8 regulates the localization and spatial interactions of the Gα_i_-GDP:LGN:NuMA complex in the cell cortex [19]. Reduction of RIC8 level slows down guanine nucleotide exchange on Gα_i_ and thereby inhibits liberation of Gα_i_-GTP and NuMA from the ternary complex, which in turn results in lower mitotic spindle motility, prolonged mitosis and mitotic arrest [20]. The same partners of afore-mentioned complex, LGN and NuMA localize to the meiotic spindle apparatus in analogy with RIC8 after germinal vesicle breakdown during maturation of oocyte [38, 39]. Earlier studies of the relative Gα_i1_ and Gα_i2_ mRNA expression have indicated that they are both maternally expressed during the mouse oocyte growth and maturation [40]. To our knowledge, the expression pattern of Gα_i_ protein in the oocyte maturation has not been mapped so far. By using antibody against Gα_i1/2_, which recognizes both Gα_i1_ and Gα_i2_ subunits, we found that Gα_i1/2_ localizes uniformly in the cytoplasm and in the cell cortex during meiosis of maturing oocyte. To some extent it was detectable also in the meiotic spindle. It was shown earlier that in the metaphase Gα_i1_ localizes in the cell cortex of HeLa cells, but Gα_i2_ is confined to the mitotic spindle [20]. Consistent with that RIC8 and Gα_i1/2_ co-localize in some regions of cell cortex of oocyte and in the meiotic spindle. Several studies have indicated that RIC8 is required for the maintenance of Gα_i_ levels and its localization to the plasma membrane [12–14]. Moreover, RIC8 also acts as a biosynthetic chaperone at the Gα subunit folding and participates in their subsequent proper membrane targeting [6, 7]. Analogously, we demonstrated that the inhibition of *Ric8* synthesis during oocyte maturation interfered with the correct localization of Gα_i1/2_ and reduced its level in the cell cortex region. However, the localization pattern of Gα_i1/2_ in the cytoplasm and the localization of LGN were not influenced by the *Ric8* siRNA treatment of oocytes. Although the downregulation of *Ric8* expression had no statistically relevant effect on the morphology of maturing oocytes, we observed a tendency for some oocytes to divide abnormally (forming two or three almost equal cells). Furthermore, meiosis I lasted longer in *Ric8* siRNA treated cells and some oocytes could not maintain the correct positioning of chromosomes in the metaphase arrests.

In addition, RIC8 functionally also interacts with the Gα_i_-GDP:RGS14 (regulator of G protein signaling-14) signaling complex and regulates its activation state [41]. Interestingly, in mammalian oogenesis, RGS14 is initially expressed in oocytes, but is degraded at the second meiotic arrest. Prior to the first mitosis, RGS14 is de novo expressed by the activated embryonic genome and it co-localizes with anastral mitotic apparatus of the zygote [28]. Remarkably, in exponentially proliferating cell culture (e.g. HeLa cells) RGS-14 localizes in the nucleus during interphase and is distributed to the centrosomes and astral microtubules during mitosis, and alteration of RGS-14 levels leads to cell growth arrest [28]. The similar expression profile of RIC8 protein during oogenesis, and in early zygote with expression and localization of its interaction partners, suggests that RIC8 might function in concert with Gα_i_, LGN, NuMA and RGS14 to regulate meiosis and mitosis.

After fertilization oocyte completes meiosis and the genetic material of mature gametes forms the paternal and maternal pronucleus. We found that RIC8 localized to the female and male pronucleus and accumulated in the nucleolus precursor bodies (NPBs). RIC8 also concentrated in NPBs of the second polar body and blastomeres of two-cell embryos. Whereas nucleolus of growing oocyte is active and mainly responsible for ribosome biogenesis, in analogy with nucleoli of somatic cells, transcription largely ceases in the nucleoli of fully grown oocytes and blastomeres of early cleavage embryos until the transition from the maternal to embryonic genome [42, 43]. The function of these “inactive” nucleoli in mature oocytes and blastomeres is not clear yet, but mouse embryos lacking nucleoli fail to develop past the first few cleavages [44]. Although the oocyte nucleolus is not needed for the progression of meiosis to the second metaphase, it is indispensable in further early development [43]. Accumulation of RIC8 in the rim region of NPBs, where methylated DNA and centromeres assemble to nucleoli [45], suggests that RIC8 may also be involved in the maintenance of nucleolar function or architecture.

We also found RIC8 expression in several regions of mouse reproductive tract, like epithelium of Fallopian tube and uterus. Interestingly, RIC8 accumulated in the basal layer of cilia in the ciliated epithelium of ampulla region. Two types of cilia, motile and primary are present in mammalian cells. The motile cilia cooperatively beat in a wave-like pattern to generate fluid flux, which is essential for pickup and transport of the ovulated cumulus-oocyte complex [46]. One of RIC8 interaction partners, Gα_i2_ [1], localizes specifically in ciliated cells of rat [47] and human [48] Fallopian tube, implying the importance of Gα_i2_ in signal transduction in the ciliary membranes. Gα_i_ proteins also couple to progesterone receptors [49], which are found on membranes of motile cilia of the mouse oviduct, where they localize to the lower half and the base of the cilium [50] and might participate in ciliary beat regulation [51]. Therefore it is reasonable to assume that RIC8 might also be involved in ciliary beat regulation in the oviduct since it amplifies the signals from G-protein coupled receptors and co-localizes in cilia with Gα_i2_.

In conclusion, we present novel data about a dynamic localization of guanine nucleotide exchange factor RIC8 in mouse oogenesis, at fertilization and initial steps of oocyte first cleavage. We demonstrated for the first time that the redistribution of RIC8 during mouse oogenesis is highly regulated and strictly follows the oocyte growth and maturation, as well as the phases of meiosis. The results of present study form a good basis for the further unraveling of the RIC8 function in gametogenesis, fertilization and early development of mammals.
